# Systemic lupus erythematosus and pregnancy: a comprehensive clinical review of maternal, fetal, and treatment considerations

**DOI:** 10.1186/s42358-026-00555-x

**Published:** 2026-06-10

**Authors:** Diego F. Wyszynski

**Affiliations:** Center of Excellence in Pregnancy and Lactation Studies, Thermo Fisher Scientific Inc, London, England, United Kingdom

**Keywords:** Systemic lupus erythematosus, Pregnancy, Adverse outcomes, Disease flare, Placental insufficiency

## Abstract

Systemic lupus erythematosus (SLE) is a chronic autoimmune disease characterized by multisystem inflammation, predominantly affecting women of reproductive age. Consequently, its impact on pregnancy outcomes represents a critical clinical concern. Advances in disease monitoring and management have reduced pregnancy-related complications; however, risks remain higher than in the general population. Adverse outcomes, including fetal loss, preterm delivery, preeclampsia, thrombotic events, and low birth weight, are particularly common in patients with lupus nephritis. Placental insufficiency and immune-mediated inflammatory mechanisms are thought to contribute to these complications. Pregnancy may also precipitate disease reactivation, although the timing and frequency of flares are variable. Higher disease activity is consistently associated with poorer outcomes, yet prediction of flares or adverse events remains challenging. In addition, certain SLE therapies pose teratogenic risks, underscoring the importance of individualized treatment planning. Optimal outcomes are most likely when conception occurs during a period of disease quiescence, with close multidisciplinary monitoring and the use of pregnancy-compatible therapies to maintain disease control.

## Introduction

Systemic lupus erythematosus (SLE) is a chronic autoimmune disease characterized by multisystem inflammation, with clinical manifestations ranging from mild dermatologic or musculoskeletal involvement to severe, life-threatening organ damage. Cardiovascular complications are common, with patients experiencing a two- to threefold increased risk of stroke and myocardial infarction compared with the general population [1]. SLE frequently coexists with antiphospholipid syndrome (APS) and the presence of antiphospholipid (aPL) antibodies [2]. Childhood-onset SLE often presents with greater systemic involvement, a more aggressive disease course, and higher mortality rates than adult-onset SLE [3, 4].

The etiology of SLE remains incompletely understood, but genetic, immunologic, environmental, and hormonal factors are all believed to contribute to disease pathogenesis [1, 5]. In genetically predisposed individuals, environmental exposures may trigger aberrant immune responses, leading to the loss of self-tolerance and subsequent tissue inflammation [6]. More recently, oxidative stress and alterations in the gut microbiota have also been implicated as potential contributors to disease development and progression [1].

SLE incidence varies globally, ranging from 0.3 to 23.2 per 100,000 person-years [7]. It is more prevalent in non-white populations [6–8] and may be more frequent in Europe and Australia than in the U.S [8], though findings are inconsistent [7]. Incidence has risen in recent years, partly due to improved diagnosis [7, 9]. Nearly 90% of patients are female [6, 10], with peak incidence occurring during reproductive years [10, 11]. As a result, the impact of SLE on pregnancy is a major concern for affected individuals. Several recent reviews have addressed SLE in the context of pregnancy; however, most have been limited in scope, either geographically [12], by focusing on specific aspects such as fertility and assisted reproductive technologies [13] or disease flares [14], or by concentrating on particular subgroups, including women with lupus nephritis (LN) [15] or co-existing antiphospholipid syndrome (APS) [16]. This review seeks to provide a comprehensive overview of the key issues related to SLE and pregnancy, encompassing maternal and neonatal outcomes as well as treatment considerations.

## How does SLE impact maternal and neonatal outcomes?

Despite improvements in monitoring and management, pregnancy in women with SLE remains high risk [17]. Approximately 25% of pregnancies end in fetal loss [10, 18–21]. While miscarriage rates are comparable to the general population [9, 22], stillbirth rates are significantly higher [11, 22]. Increased SLE activity, particularly in early pregnancy, elevates the risk of fetal loss [10, 18, 21], with proteinuria, thrombocytopenia, and hypertension being independent risk factors [18]. APS further increases the likelihood of pregnancy loss, particularly after 20 weeks of gestation [10, 18, 20]. Indeed, recurrent pregnancy loss is an important clinical criterion for the diagnosis of APS [23]. Women with a history of three or more previous miscarriages have a higher likelihood of experiencing a miscarriage in subsequent pregnancies [9].

Preterm delivery occurs in 20–71% of SLE pregnancies [9–12, 17, 19, 21, 24, 25]. The elevation of the risk of prematurity is partly explained by an increased incidence of premature membrane rupture [5, 12, 24]. In addition, a sizeable proportion of premature deliveries are induced in order to protect the health of the mother and/or the baby [19, 24], suggesting that spontaneous and induced preterm delivery may have common risk factors [24]. These are similar to those for pregnancy loss, and include aPL antibodies [9, 20], hypertension [9, 20], and disease activity, whether measured by clinical index [10], medication dose [19] or serum C3 complement level [20].

Preeclampsia is a condition unique to pregnancy and is thought to arise from vascular dysfunction in the placenta. Severe forms of it are characterized by very high blood pressure and proteinuria, and may also be accompanied by thrombocytopenia, hemolysis, renal insufficiency, and liver involvement [26]. The risk of developing preeclampsia is increased from around 5–8% in the general population [22] to 13–52% in SLE pregnancies [11, 12, 17, 21, 27]. The highest risks for preeclampsia are seen in women with pre-existing hypertension [9], disease activity during pregnancy [12], low C3 levels [9], and lupus nephritis [21, 28]. Distinguishing between preeclampsia and an LN flare can be extremely challenging, particularly as they can co-exist in the same patient, but is vital as management strategies differ significantly [23, 29]. Both present with proteinuria, hypertension, and edema of the lower extremities, as well as possible systemic effects [11, 22, 23, 29]. While preeclampsia will resolve after delivery of the fetus, active LN requires immunosuppressive therapy [22, 28, 29].

Thrombosis risk increases during normal pregnancies, due partly to compression of the vena cava by the enlarging uterus and the mother’s relative immobility, both of which may cause venous stasis in the lower limbs. In addition, changes in the maternal clotting system which prevent massive blood loss during delivery, are also influential. The presence of SLE, particularly cases with positivity to aPL antibodies, increases the risk of thrombotic events still further [29]. APS is a known cause of recurrent arterial or venous thrombosis and may explain the higher incidence of deep vein thrombosis and pulmonary embolism [9, 17], and stroke [17] that occur during SLE pregnancies.

Other adverse maternal outcomes encountered more frequently in pregnant patients with SLE include antepartum and postpartum hemorrhage [9, 17, 25]; gestational diabetes mellitus [11]; postpartum infections, including sepsis and pneumonia [17]; and cesarean section [9, 11, 12, 17, 25]. Furthermore, the overall risk of maternal death is reported to be 20-times higher than in the non-SLE population [17]. The increased incidence of infection is likely due to both SLE-related immune dysfunction and immunosuppressive therapy [17]. Although low C3 is predictive for cesarean section [9], no other clear risk factors have emerged.

Assessing the risk of low birth weight in SLE pregnancies is complicated by the high rate of preterm births. Therefore, an assessment of weight according to gestational age is usually made, with babies being classified as small for gestational age (SGA) if they weigh less than the tenth percentile compared to national standards [22]. Even so, as much of the fetal growth occurs in the final weeks of gestation, impaired fetal growth will not necessarily manifest in pregnancies that have completed early [10]. Given this, it is perhaps not surprising that the rate of SGA births in SLE cohorts is usually reported to be similar to that experienced by the general population, at around 10% [19], although much higher rates have been reported [10, 11, 21, 25]. Fetal growth restriction has also been reported to be more common in SLE pregnancies [11]. No consistent risk factors for SGA in patients with SLE have been identified, although disease activity may be influential [10].

Babies born to mothers with SLE are more likely to be admitted to a neonatal intensive care unit [9, 11], and this is often due to respiratory distress, neonatal sepsis, or prematurity [9]. The rates of perinatal deaths [11, 12], low 5-minute Apgar scores and neonatal infections are also increased in the offspring of patients with SLE [11]. Fetal distress and asphyxiation immediately following birth have also been reported and appear to be more common when the mother has been diagnosed with childhood-onset SLE [25].

Neonatal lupus erythematosus (NLE) is a rare congenital autoimmune condition caused by the passive transfer of maternal antinuclear IgG class antibodies across the placenta to the fetal circulation [13, 30]. These antibodies begin to cross the placenta around the 12th week of gestation and target intracellular ribonucleoproteins, leading to hepatic, hematological, and cutaneous abnormalities in the fetus. However, these manifestations typically resolve shortly after birth as maternal antibody levels in the infant’s circulation decline [13]. NLE has been consistently linked with maternal positivity for Sjogren’s syndrome autoantigen type A (Ro/SSA) or B (La/SSB) autoantibodies (aAbs) and with a diagnosis of maternal SLE [5, 28, 30], although around half of all mothers are asymptomatic [30].

Of the many clinical manifestations of NLE, congenital heart block is the commonest and most significant [23]. It is usually detected in the second trimester in an otherwise normal fetus, in the absence of structural cardiac abnormalities [2]. The precise mechanism of causation is unclear, but it is possible that maternal aAbs bind with autoantigens in the fetal heart or influence the electrophysiology of the developing heart by cross-reacting with other molecules on the surface of fetal cardiac cells [31]. However, the finding that congenital heart block may develop in one, but not both, fetuses in monozygotic twin pregnancies, suggests that its etiology depends on many factors [32]. Over three-quarters of infants with the condition develop third-degree, or complete, heart block, which has a high mortality rate and often requires the permanent implantation of a pacemaker [30].

While all pregnancies in patients with SLE should be considered high-risk, this is particularly true for patients with LN. This condition is associated with a lower rate of live births and risks of gestational hypertension, preeclampsia, proteinuria, hypocomplementemia, preterm birth and low birthweight over and above those seen in SLE patients without renal involvement [12, 22, 28, 33]. It is also associated with an increased risk of SLE reactivation [22]. However, LN does not significantly influence the risk of spontaneous abortion, stillbirth, cesarean section, or positivity for APS or aPL [28]. Rare cases of LN are diagnosed during pregnancy, usually in association with SLE activity. Proliferative LN in pregnancy requires urgent treatment, particularly if renal function is impaired. In severe cases, medical abortion is indicated [2].

Similarly, the coexistence of APS and SLE is associated with an increased risk of adverse pregnancy outcomes. Patients with both conditions have consistently higher rates of thrombotic events, preeclampsia, and fetal growth restriction. This subgroup may benefit from intensified anticoagulation and immunosuppressive treatment regimens [16].

The ability to predict which SLE patients will experience a poor pregnancy outcome would significantly improve the care of this high-risk population, but it remains a challenge. In addition to the associations between complement levels and the outcomes already discussed above, several other biomarkers may indicate an increased risk of adverse pregnancy outcome. Low expression of erythrocyte complement receptor 1 in the first and second trimesters has shown a significant association with a composite endpoint consisting of fetal and neonatal death, preterm delivery, and SGA baby [27]. Other biomarkers that may help to predict adverse pregnancy outcomes include elevated levels of soluble endoglin and fms-like tyrosine kinase-1(sFlt-1), low levels of placental growth factor (PIGF), and the ratio between sFlt-1 and PIGF [34, 35]. Experiments with machine learning methods to predict adverse pregnancy outcomes in patients with SLE have not offered clear benefits over regression-based approaches and it is likely that significant improvements will not be achieved until new biomarkers and risk factors have been identified [36].

### How does pregnancy influence SLE?

Pregnancy’s impact on SLE activity is debated. This may be partly due to the fact that the signs and symptoms of pregnancy, which include severe fatigue, post-partum hair loss, increased shortness of breath, arthralgias, skin rash, and headaches, can be mistaken for signs of active SLE [22, 29]. However, a diagnosis of lupus can be confirmed by a blood platelet count below 100,000, decreased C3 and C4 complement levels, or a rise in the level of anti-double stranded DNA (anti-dsDNA) antibodies [22].

During pregnancy, the synthesis of Th2 cytokines is increased while Th1 cytokines are inhibited. SLE is characterized by the overproduction of autoantibodies that may mediate organ damage, and is therefore a Th2 disease. This contrasts with the typical Th1 mechanism seen in conditions like rheumatoid arthritis (RA), where joint damage is cell-mediated. Thus, the expectation would be for pregnancy to have a beneficial effect on the course of diseases such as RA but a detrimental one on SLE [29].

Early studies report low levels of disease exacerbation during pregnancy [37, 38]. However, larger, more recent studies have demonstrated that pregnancy is associated with relatively high levels of SLE disease activity and the occurrence of flares [10, 39]. The current thinking is that measurable SLE activity will occur in up to 70% of pregnancies [20, 22, 25, 39], although the risk of a moderate to severe flare is much lower [10, 39].

For most patients, these flares are not serious. Skin, joint, and blood disorders are frequently reported [25, 40], and while these symptoms may make pregnancy uncomfortable, the long-term impact is minimal. However, in a minority of patients, there is a dramatic worsening of symptoms that may be life-threatening [22]. Severe flares include pleuritis, arthritis, thrombocytopenia, cerebritis, myositis and pericarditis [39]. Regardless of their severity, the number of flares experienced is positively associated with damage accrual [41]. Patients experiencing their first onset of SLE while pregnant typically experience more severe disease and have a higher prevalence of LN and severe thrombocytopenia than those diagnosed outside of pregnancy [42].

No consistent pattern for the timing of SLE flares during pregnancy has emerged and they can occur in any trimester, although it is extremely unusual for flares to occur consistently throughout any one pregnancy [10]. It is important to note that the risk extends past pregnancy [20, 43], with approximately half of all flares occurring in the first few months following delivery [20]. Given the complexity of SLE pathogenesis and the heterogeneity among pregnant patients with SLE, the optimal strategy for minimizing the risk of disease flares during pregnancy remains uncertain [14].

Low levels of the complement factors C3 and C4 are reliable predictors of disease activity, even if the changes are not clinically relevant. While it has been suggested that C4 is important in flare initiation and C3 level is associated with the actual tissue damage during a flare [44], elsewhere it has been reported that it is the level of C3 that appears to be particularly important in predicting flares: the less pronounced the increase in C3 levels from conception throughout the first trimester of pregnancy, the higher the risk of a subsequent disease flare. This risk is even higher in patients with LN [33], which is itself an established risk factor for disease activity during pregnancy [25, 28]. The interaction between these two variables may explain the increased rates of both SLE and renal flares observed in LN patients [14, 25]. Renal flares can be particularly aggressive, sometimes resulting in acute renal failure and even maternal death [23]. Additional risk factors for disease flares include younger maternal age, baseline thrombocytopenia, positive anti-dsDNA antibody status, hypertension, and baseline disease activity, whether assessed clinically or inferred from medication use [14]. Discontinuation of chloroquine treatment and a history of more than three flares before pregnancy, particularly if these were severe, are also associated with the occurrence of flares [20]. Additionally, patients with childhood-onset SLE are more likely to experience disease activity during pregnancy than those diagnosed in adulthood [25].

## Mechanisms of adverse pregnancy outcomes in SLE

The mechanisms by which SLE affects pregnancy outcome have not yet been fully elucidated. However, many of these adverse events are associated with impaired placentation and this is likely to play a major role. In the early stages of pregnancy, trophoblast cells from the placenta invade the wall of the uterus, where they transform the uterine spiral arteries into wide channels to allow low-resistance blood flow to the placenta. If the invasion of cells is inadequate, the spiral arteries may not enlarge sufficiently, and poor placental blood flow may occur throughout the pregnancy. Vascular insufficiency in the placenta may be sufficient to cause the higher rate of spontaneous preterm births associated with SLE pregnancies. Poor placentation and dysfunctional angiogenesis are also known to increase the risk of preeclampsia [26].

The development of the placenta is regulated by several mediators, including the glycoproteins syncytin-1 and syncytin-2. Their main role is to promote the formation of the syncytiotrophoblast, which divides maternal and fetal tissues, by inducing cell-cell fusion of single nucleated cytotrophoblast cells [45, 46]. Thus, they are involved in proliferation and survival, as well as homeostasis and differentiation, of the syncytiotrophoblast. Furthermore, the immunosuppressive domains (ISDs) of syncytin-1 and syncytin-2 have immunomodulatory properties which induce maternal tolerance of the fetus. A failure to establish the immunoregulatory function of the syncytiotrophoblast can result in pregnancy complications [46].

The genes and transcripts of both syncytin-1 and syncytin-2 correspond to human long non-coding RNAs (lncRNAs), three of which have been found to be dysregulated in both SLE patients and non-SLE women with high-risk pregnancies. Furthermore, protein-coding genes adjacent to the lncRNAs have been reported to be associated with gynecological or obstetric complications and with SLE risk. It has been suggested that abnormal nuclear and cytoplasmic expression of lncRNAs in the syncytiotrophoblast and placental tissue of pregnant women with SLE may prevent the transcription and translation of syncytin mRNA, as well as locally reducing maternal immune tolerance to embryonic tissues by sequestering syncytin transcripts and preventing surface expression of the ISD. Alternatively, syncytin transcripts may align with lncRNA regulatory sequences and interfere with the transcription of neighboring genes which have already been shown to be important in the development of SLE or adverse obstetric outcomes [47].

Several other processes may also lead to placental issues. For instance, the higher incidence of thrombosis and other vascular pathology observed in the placentas of pregnant women with SLE [28, 48] suggests that reduced uteroplacental perfusion may restrict fetal growth, leading to pregnancy loss or the birth of SGA infants [5, 22, 28]. Alternatively, SLE patients with LN and high blood pressure may experience compromised microcirculation in the uteroplacental blood vessels, with pregnancy itself contributing further to renal impairment [28]. Placental blood flow may also be affected by the presence of excess glucocorticoids, which are often used in the treatment of SLE. They are key mediators of vascular resistance in the systemic circulation and thus may influence vascular resistance in the placenta and exert an effect on fetal growth [49].

Animal studies have demonstrated that complement activation causes dysregulation of the angiogenic factors required for normal placental development, leading to fetal loss and IUGR. Functional deficiency of vascular endothelial growth factor (VEGF) and elevated levels of soluble VEGF receptor 1 are associated with placental deficiencies, which can be reversed if complement activation is inhibited [50]. The human placenta experiences complement-mediated immune attack at the maternal-fetus interface. In normal pregnancies, complement activation is controlled by an inhibitory protein [51]. However, in patients with SLE, excessive complement activation overwhelms regulatory pathways. Furthermore, the infiltrating leukocytes recruited and activated by complement may result in an excess of anti-angiogenic factors leading to impaired placental development [52]. In patients with coexisting APS, complement activation is triggered by antiphospholipid antibodies, leading to inflammation, thrombosis, and impaired blood flow. This disruption of normal placental function contributes to adverse pregnancy outcomes [53].

The level of anti-dsDNA antibodies, which fluctuates with disease activity, may also be relevant. The observation of increased positivity for anti-dsDNA antibodies in pregnant patients with childhood-onset SLE than those diagnosed in adulthood suggests that childhood SLE may be more aggressive during pregnancy. This is borne out by the higher rates of adverse maternal and neonatal outcomes reported in this group of patients. Although the pathogenesis of the difference between childhood- and adult-onset SLE remains unknown, genetic factors are thought to be important, and it is speculated that these could also influence pregnancy outcomes [25].

Finally, inflammation associated with active SLE has been implicated in multi-organ damage, with the kidneys being particularly susceptible due to their high vascular perfusion demands [12]. SLE-related inflammation may also mimic the effects of chorioamnionitis, promoting amniotic sac disruption, cervical ripening, and uterine contractions, ultimately increasing the risk of preterm birth [22].

## Treatment of SLE during pregnancy

Although women are advised to defer conception until disease activity has been controlled for at least six months, a substantial proportion of pregnancies still occur during periods of active disease. In such cases, pharmacologic therapy may be required to prevent or manage SLE flares [22]. Certain agents commonly used in SLE, including cyclophosphamide, mycophenolate mofetil, and leflunomide, are contraindicated during pregnancy due to established or potential teratogenicity and their association with high rates of pregnancy loss [22, 23, 54]. Furthermore, women who have been treated with methotrexate prior to conception are advised to receive folic acid supplementation to mitigate the risk of neural tube defects, given the drug’s inhibitory effect on folate metabolism [22]. Other medications, including apremilast, avacopan, baricitinib, bosentan, filgotinib, leflunomide, mepacrine, tofacitinib, upadacitinib, voclosporin, and the biologics anifrolumab, eculizumab, guselkumab, mepolizumab, and Risankizumab, should be avoided during pregnancy because of insufficient safety data, rather than definitive evidence of teratogenicity [55]. The European League Against Rheumatism (EULAR) considers sulfasalazine safe for use in pregnancy [55]; however, it has been associated with drug-induced SLE in some reports [56], although this association is not consistently observed [57]. Consequently, its use in the management of SLE remains limited.

Treatment strategies for SLE have evolved in recent years, shifting from conventional immunosuppressive regimens toward more targeted therapies designed to reduce both disease activity and treatment-related complications [57]. In pregnant patients, the potential risks of pharmacologic therapy to the fetus must be carefully balanced against the risks of uncontrolled maternal disease. Treatment selection should ultimately be guided by a shared decision-making process between the patient and her healthcare providers. The therapeutic goal in SLE is to achieve remission or maintain low disease activity [55]. The pharmacologic options that may be considered to achieve these goals in pregnant patients with SLE are outlined below.

### Antimalarials

Hydroxychloroquine (HCQ) is an antimalarial medication widely used in the treatment of SLE [28]. By inhibiting the synthesis of reactive oxygen species, it acts as an antioxidant, thereby preventing auto-oxidative tissue damage at sites of inflammation [58]. In addition, HCQ inhibits endosomal Toll-like receptor activation and calcium signaling [59]. HCQ is able to cross the placenta and as cord blood concentrations mirror those found in maternal blood, this suggests that the level of exposure to HCQ during pregnancy is similar in the mother and fetus [60]. This raises the possibility of potential teratogenic and toxic effects on the fetus. Because these events are most likely to occur during the first 12 weeks of pregnancy, some have suggested delaying the initiation of hydroxychloroquine (HCQ) treatment until after this period [16]. However, studies in pregnant populations indicate that HCQ is very well tolerated and no elevation in the rate of adverse pregnancy outcomes or fetal anomalies has been observed following its use [22, 61]. While high doses of chloroquine have been linked to the development of ocular or auditory defects [22, 62] and oral clefts, respiratory anomalies, and urinary defects [63], no visual, hearing, growth or developmental abnormalities were observed in a series of over 100 infants born to women treated with HCQ during pregnancy [60].

HCQ treatment also reduces the risk of preterm delivery [64] and congenital heart block (CHB) in neonates [65]. In women with a prior history of fetal congenital heart block (CHB), many clinicians consider initiating hydroxychloroquine (HCQ) before a subsequent pregnancy [66]. In adults, HCQ reduces the risk of systemic lupus erythematosus flares [67], preeclampsia and gestational hypertension [64], and is associated with improved renal outcomes in LN [68]. The observed association between HCQ use and a lower risk of preeclampsia [64] may be attributable to its protective effects against inflammation and endothelial injury within the placenta [69]. It may also help to prevent the thrombotic effects of aPL antibodies [70], and its use has been linked to a reduced risk of maternal deaths, both from SLE and other causes [71]. Pregnant SLE patients treated with a combination of HCQ and aspirin are more likely to have a full-term pregnancy and a correspondingly lower risk of preterm birth, as well as reduced risks of hypertension and pregnancy loss. Neonates have significantly higher birth weights and 1- or 5-minute Apgar scores and lower rates of neonatal asphyxia [72].

### Tacrolimus

Tacrolimus inhibits T-cell activation, which contributes to the initiation and perpetuation of autoimmunity in SLE [73], by reducing calcineurin activity and subsequent transcription of the gene encoding interleukin-2 [74]. It has been reported to cross the placenta [75]. Furthermore, calcineurin inhibitors, including tacrolimus, have been implicated in the initiation of endothelial damage [76], which is directly related to placental vascular abnormalities [77]. Therefore, the use of tacrolimus during pregnancy could theoretically be associated with an increased frequency of adverse outcomes [74]. Furthermore, tacrolimus has been associated with reduced renal function in patients receiving it for more than three months [78] and has also induced insulin resistance in animal studies [79], both of which may increase the risk of adverse pregnancy outcomes. However, administration of tacrolimus to pregnant patients has not resulted in the deterioration of blood pressure, glucose metabolism, or renal function and there is no increase in the frequency of adverse pregnancy outcomes [74, 80]. When tacrolimus is combined with aspirin, a lower frequency of preeclampsia, hypertensive pregnancy disorders, and low birth weight infants has been reported [74]. This finding may be due to the anti-inflammatory properties of aspirin in conjunction with its effect on platelet aggregation [24].

### Cyclosporine

Similar to tacrolimus, cyclosporine is a calcineurin inhibitor [54] and is widely used as an immunosuppressive agent in the management of autoimmune diseases, including SLE. In pregnancy, disease flares occur in only a minority of patients, and the prevalence of maternal and fetal complications appears comparable to that of the general population [81]. Nonetheless, cyclosporine use has been associated with an increased risk of gestational hypertension and preeclampsia [82]. Despite these potential risks, EULAR considers cyclosporine safe for use during pregnancy [55].

### Azathioprine

Among the available immunosuppressive agents, azathioprine (AZA) is considered the safest during pregnancy, as the fetal liver lacks the enzyme required to convert the drug into its active metabolite [22]. Nevertheless, both transplacental and transamniotic transfer of AZA and its metabolites have been documented [83]. Although teratogenic effects have been observed in animal studies [23], women with low SLE activity who continue AZA throughout pregnancy generally experience favorable maternal and fetal outcomes [61]. Consequently, discontinuation during gestation is typically not necessary [22]. In contrast, AZA use in the context of severe, active SLE has been associated with an increased risk of pregnancy loss, although available data do not suggest an elevated risk of congenital malformations [84]. A slightly increased risk of childhood infections following in utero or breastfeeding exposure to AZA has also been reported, although the underlying mechanism remains unclear [61].

### Colchicine

Colchicine is an anti-inflammatory agent employed in the management of various rheumatologic conditions [85]. In SLE, it is primarily indicated for the treatment of cardiac manifestations, particularly pericarditis, where its use in combination with nonsteroidal anti-inflammatory drugs (NSAIDs) or corticosteroids significantly reduces both initial episodes and subsequent recurrences [86]. Use of colchicine during pregnancy has not been associated with an increased risk of major congenital anomalies, and miscarriage rates are significantly lower in women receiving colchicine compared with those who do not. However, birthweight and gestational age are, on average, lower than in healthy pregnant women [85]. Despite these findings, EULAR considers colchicine safe for use during pregnancy [55].

### Biologics

The use of biologic agents in the management of SLE has expanded substantially in recent years, accompanied by a growing body of evidence regarding their efficacy and safety. Prior to initiating any biologic, its potential to achieve or maintain disease quiescence should be carefully weighed against the risk of relapse if the drug is withheld, as well as the likelihood of transplacental transfer [55].

Current evidence indicates that tumor necrosis factor inhibitor (TNFi) biologics are not associated with an increased risk of congenital malformations, miscarriage, preterm birth, low birth weight, preeclampsia, or severe neonatal infection, and may therefore be considered safe for use during pregnancy [55, 87, 88]. For non-TNFi biologics, the available data are more limited; however, reported rates of adverse pregnancy outcomes do not appear to be appreciably higher than those observed in the general population [55, 89].

Belimumab is a recombinant human IgG-1λ monoclonal antibody that inhibits B-cell activating factor (BAFF). Excess BAFF expression promotes the survival of autoreactive B cells and the production of autoantibodies. By binding to soluble BAFF, belimumab blocks its interaction with B-cell receptors, thereby reducing B-cell survival [57]. Approved for the management of SLE [90], it has been used in a small number of pregnant patients. Belimumab has demonstrated efficacy in controlling active disease, is generally well tolerated [91], and has not been associated with fetal anomalies [90, 92]. The greatest therapeutic benefit appears to occur in patients with high disease activity and limited organ damage [57]. Current EULAR recommendations support its use in patients who do not respond adequately to hydroxychloroquine, or as a means to facilitate corticosteroid tapering or discontinuation [93].

### Corticosteroids

Many patients with mild SLE activity are treated with low-dose corticosteroids, usually prednisone. The main side-effects of this treatment are an increased risk of hypertension and diabetes [22]. Systemic corticosteroid use has been associated with a doubling of the risk of cleft lip or palate, although the absolute risk is very low [94]. Higher doses of corticosteroids can be used to combat moderate SLE activity. While prednisone and prednisolone only cross the placenta in small amounts, fluorinated glucocorticoids, such as dexamethasone and betamethasone, are easily transferred [23, 95]. Although these steroids can be used to ensure fetal lung maturity before a preterm delivery, they have also been associated with high levels of adverse pregnancy outcomes [95] and thus are not recommended for use during pregnancy unless the intention is to treat the fetus [22, 23, 29]. When prednisone or prednisolone is required during pregnancy, the dose should be tapered to less than 5 mg/day whenever possible [55].

### Non-steroidal anti-inflammatory drugs

NSAIDs can be used safely throughout the second trimester of pregnancy [22], although isolated cases of oligohydramnios have been reported following administration [96, 97]. However, they should be avoided in the earliest stages of pregnancy, due to an increased risk of miscarriage, thought to be caused by NSAID-induced prostaglandin inhibition due to the drugs interfering with implantation and placental circulation [98]. NSAIDs should also be avoided in the third trimester, as they can prolong labor and may promote the constriction or premature closure of the ductus arteriosus [99]. A significant association between antenatal NSAID exposure in the third trimester and neonatal pulmonary hypertension, which appears to be dose-related, has also been demonstrated [100]. However, there is no evidence of an increased risk of congenital abnormalities [101].

The most recent EULAR guidelines state that corticosteroids and NSAIDs may be used during pregnancy when necessary to control active disease [55]. Earlier guidelines have recommended that pregnant patients with systemic lupus erythematosus (SLE) who are at increased risk of preeclampsia, particularly those with LN or positive aPL, should be offered low-dose aspirin (LDA) during gestation. Patients with APS, whether SLE-associated or primary, should receive combination therapy with LDA and heparin to reduce the risk of adverse pregnancy outcomes [54]. However, emerging evidence regarding optimal dosing and timing supports a more restricted use of NSAIDs in pregnant patients. Where feasible, disease control should be achieved by adding or switching to pregnancy-compatible conventional or biologic disease-modifying antirheumatic drugs (DMARDs) [55].

### Intravenous immunoglobin

IVIg has been associated with significant reductions in SLE disease activity [102], particularly in refractory cases [1] and in patients with renal involvement [103]. Its proposed therapeutic effect is thought to result, at least in part, from antioxidant properties, although this mechanism has not been definitively established [1]. Data on IVIg use during pregnancy are limited; however, reports in non-pregnant populations indicate few adverse reactions and no congenital abnormalities [22, 103]. IVIg is therefore considered a relatively low-risk therapy [104]. EULAR recommends restricting its use during pregnancy to cases of severe, refractory disease [55].

### Future developments

Dapirolizumab pegol (DZP) is a PEGylated anti-CD40L antibody fragment that inhibits CD40–CD40L interactions, which are critical in T cell–dependent B cell responses and autoimmune inflammation in SLE. By omitting the Fc domain, DZP retains therapeutic efficacy without triggering platelet activation [57]. Early clinical trials suggest that DZP improves disease activity in patients with SLE and has an acceptable safety profile [105, 106]. Its PEGylated structure may limit placental transfer, potentially making it suitable for use during pregnancy, although further safety data are needed [57].

In addition, CD19-directed chimeric antigen receptor (CAR) T cell therapy, originally developed for B cell malignancies, represents a novel approach for treating severe SLE. CAR T cells are engineered to express receptors that target CD19 on B cells, leading to profound B cell depletion. Given the central role of B cells in SLE pathogenesis, this therapy offers the potential to eliminate pathogenic autoantibodies and restore immune tolerance, particularly in patients who are refractory to conventional treatments. Preliminary data suggest a more favorable safety profile in patients with SLE compared to those with B cell malignancies [107], although further studies are necessary to define its role in clinical practice. Data on pregnancy following CAR-T cell therapy are limited to a small number of case reports, which to date have not identified any significant safety concerns [108–110].

T cell engager therapy, which relies on activated T cells to selectively destroy pathogenic immune cells, presents a promising alternative to CAR T cell therapy. It offers several advantages, including a simpler manufacturing process, lower cost, no need for lymphodepleting chemotherapy, and the ability to adjust dosing based on clinical response [57]. However, data on its use during pregnancy are currently unavailable.

Given the links between SLE and oxidative stress and the gut microbiota, other future therapies may revolve around controlling these factors through methods such as pathobiont restriction, specific diets, and supplementation with regulatory metabolites [1].

A major limitation in the current management of SLE is the inability to account for the underlying molecular heterogeneity that drives disease activity. Precision medicine offers the potential to address this challenge by tailoring therapies to individual patient characteristics and pathogenic mechanisms. Advances in understanding the genetic variations associated with SLE may enable earlier identification of individuals at risk and inform more personalized therapeutic strategies. The identification of organ-specific genetic markers could further refine treatment by targeting therapies to those most likely to benefit. This remains a critical objective, given the substantial variability in treatment response among patients [57].

### Guidelines for the management of pregnancy in women with SLE

Due to the increased risks of an adverse pregnancy outcome, EULAR has developed a series of recommendations regarding the management of pregnancy in patients with SLE [54, 55]. Preconception counselling is strongly recommended; ideally, this should take place as soon as possible after diagnosis. Contraceptive counseling should be provided, particularly to emphasize the importance of avoiding pregnancy during periods of high disease activity and/or while receiving teratogenic medications. The potential need for treatment adjustments in anticipation of, or in response to, pregnancy should also be addressed [55]. Risk stratification, based on disease activity, LN history, and presence of aPS antibodies or APL, may help to identify potential pregnancy complications [54].

Throughout the pregnancy, regular assessment of disease activity, by monitoring of renal function parameters and serological markers, should assist in identifying disease flares and the possibility of subsequent adverse outcomes. As well as routine ultrasonographic screening during the first and second trimesters of pregnancy, additional screening is recommended at monthly intervals during the third trimester to identify fetuses with IUGR or other factors indicating a high risk of a poor outcome. This can help to determine the optimal timing for delivery, thus reducing perinatal morbidity and mortality. Fetal echocardiography is indicated in cases of suspected fetal dysrhythmia or myocarditis, particularly in the presence of maternal anti-Ro/SSA or anti-La/SSB antibodies. In this last group, the aim should be to perform echocardiography from the 16th week of gestation in all women, regardless of the presence of congenital heart block in previous pregnancies [54].

In patients with LN, kidney biopsy should be considered in the presence of progressive renal impairment and/or significant proteinuria, as it provides valuable diagnostic, therapeutic, and prognostic information [15]. Biopsy can aid in differentiating between an LN flare and preeclampsia, two conditions with overlapping clinical features but distinct management strategies [111]. However, the timing of the procedure must balance the risks and benefits to both mother and fetus. Current recommendations suggest performing kidney biopsy before 25 weeks of gestation, while avoiding the procedure after 28 weeks to reduce the risk of bleeding and preterm delivery [111].

Disease flares should be managed promptly, particularly in patients with LN. In cases of severe flares occurring early in pregnancy, therapeutic abortion may be considered. For flares later in gestation, treatment must strike a balance between effective disease control and continuation of the pregnancy [15]. Medication regimens may require adjustment to avoid agents with known teratogenicity, and a personalized treatment approach, guided by the latest evidence and patient-specific factors, should be adopted to optimize maternal and fetal outcomes [12, 15].

Low-dose aspirin (LDA) is recommended for women at high risk of preeclampsia, while both LDA and low-molecular-weight heparin (LMWH) are advised for patients with SLE-associated antiphospholipid syndrome (APS) to reduce the risk of adverse obstetric outcomes [16, 54]. However, the optimal dosing regimen for LMWH in this context has not yet been clearly defined [16]. Given the complex interplay between SLE and pregnancy, a multidisciplinary care approach is often essential to achieve the best possible outcomes for both mother and child [12, 15].

## Limitations of this review

This review specifically addresses the relationship between systemic lupus erythematosus (SLE) and pregnancy, focusing on the impact of the disease on maternal and neonatal outcomes and its management during gestation. Topics related to fertility, breastfeeding, and paternal outcomes fall outside the scope of this review and have not been addressed.

A substantial proportion of the data presented, particularly regarding pregnancy outcomes and drug safety, derives from retrospective studies, which are inherently susceptible to bias, including recall and misclassification. As a result, the true magnitude and nature of the relationship between SLE and pregnancy may be underestimated or obscured.

## Conclusions

SLE predominantly affects women of reproductive age, making its interplay with pregnancy a critical clinical concern. Pregnancy is strongly discouraged in patients with irreversible end-organ damage, pulmonary hypertension, significant restrictive lung disease, severe heart failure, or in those unable to discontinue teratogenic medications [13]. While advances in disease management have substantially reduced pregnancy-related complications for most women with SLE, notable risks persist. High disease activity at conception or in early pregnancy is associated with an increased likelihood of adverse maternal and fetal outcomes; therefore, pregnancy should be planned during periods of sustained disease quiescence.

Disease activity can increase at any stage of gestation, underscoring the importance of early recognition and prompt treatment of flares. Medication regimens should be tailored to include only agents with established safety profiles in pregnancy. Even low disease activity at conception does not preclude the possibility of severe flares later in pregnancy.

Close monitoring is essential and is best achieved through a multidisciplinary team comprising obstetricians, rheumatologists, and other relevant specialists. With appropriate management and coordinated care, many women with SLE can achieve favorable pregnancy outcomes.

## Data Availability

No datasets were generated or analysed during the current study.
